# Potential Association of Body Constitution with the Prognosis of IgA Nephropathy: A Long-Time Follow-Up of 203 Cases in China

**DOI:** 10.1155/2019/6289478

**Published:** 2019-06-23

**Authors:** Linchang Liu, Zhiwei Yin, Jiwei Ma, Shuwei Duan, Xiangmei Chen

**Affiliations:** ^1^Department of Nephrology, Chinese People's Liberation Army General Hospital (301 Hospital), Chinese People's Liberation Army Institute of Nephrology, State Key Laboratory of Kidney Diseases, National Clinical Research Center for Kidney Diseases, Beijing Key Laboratory of Kidney Diseases, Beijing 100853, China; ^2^Department of Rheumatology and Nephrology, Zhejiang Integrated Traditional and Western Medicine Hospital, Hangzhou 310003, China

## Abstract

**Objective:**

This study investigated the association between body constitution (BC) and the prognosis of IgA nephropathy.

**Methods:**

We analyzed 203 biopsy-diagnosed IgA nephropathy patients, who were followed up for (63.9±16.2) months. The participants' BC statuses were evaluated with the Constitution in Chinese Medicine Questionnaire; the relationships between clinical parameters and renal outcomes were analyzed by Cox regression.

**Results:**

Patients were classified into chronic kidney disease stages with 43.4% in stage 1, 27.1% in stage 2, 26.1% in stage 3, 3.5% in stage 4, and none in stage 5. Qi-deficiency BC type was the most common BC type in IgA nephropathy patients. In univariate analysis, proteinuria of more than 1g/d, hypertension, renal impairment (estimated glomerular filtration rate <60 mL/min), hypoproteinemia, hyperuricemia, Yang-deficiency BC, and blood-stasis BC were associated with poor prognosis. Multivariate analysis identified that hypertension (hazard ratios (HR) 3.5,* P*=0.009), renal impairment (HR 5.8,* P*<0.001), Yang-deficiency BC (HR 2.3,* P*=0.041), and blood-stasis BC (HR 2.5,* P*=0.017) were independent predictors of unfavorable renal outcomes.

**Conclusions:**

Most patients of IgA nephropathy were biopsied at an early stage. Yang-deficiency BC and blood-stasis BC at biopsy were most closely associated with the worse prognosis of IgA nephropathy along with hypertension and renal impairment.

## 1. Introduction

Body constitution (BC), a distinct characteristic of an individual in Traditional Chinese Medicine (TCM), can affect the progression of diseases [1, 2]. Yang-deficient BC is associated with the prognosis of diabetic nephropathy [3], Yang-deficient BC can be identified as a potential predictor of early HIV-related mortality, and side effects in patients initiated highly active antiretroviral therapy [4]. Blood-stasis BC is associated with peripheral arterial disease in patients with type 2 diabetes [5]. However, the role of BC in the long-term prognosis of IgA nephropathy is unclear.

IgA nephropathy is the most common primary glomerulonephritis worldwide [6]. The clinical course of IgA nephropathy is highly variable, ranging from asymptomatic microscopic hematuria to end-stage renal disease (ESRD) [7]. Previous studies have described some risk factors in the prognosis of IgA nephropathy [8, 9]. We investigated the association between BC and the prognosis of IgA nephropathy.

## 2. Materials and Methods

### 2.1. Subjects

From May 2010 to October 2014, a total of 350 patients newly diagnosed for IgA nephropathy were enrolled in this study within three days before and after renal biopsy in the Department of Nephrology, Chinese People's Liberation Army (PLA), General Hospital, and 203 cases were followed for more than 48 months. These 203 cases composed the study group, who came from seven provinces or municipalities of Northern China (Neimenggu, Hebei, Henan, Shanxi, Shandong, Beijing, and Tianjin). The inclusion criteria were as follows: (i) biopsy-proven primary IgA nephropathy; and (ii) age >14 years. The exclusion criteria were as follows: (i) IgA nephropathy secondary to chronic liver disease, henoch-schonlein purpura, systemic lupus erythematosus, and other autoimmune disorders; (ii) accompanied with acute interstitial nephritis or diabetes mellitus; and (iii) TCM therapy within the past 2 months. From May 2010 to December 2010, 64 subjects of Chinese college students and community residents without chronic disease in Beijing city were enrolled as the control group. All of the research procedures were approved by the Ethics Committee of the General Hospital of the Chinese PLA, and all subjects had signed an informed consent.

### 2.2. Clinical Information

The baseline data at the time of renal biopsy were collected. This information included sex, age, lifestyle behaviors (smoking, alcohol drinking, and less exercise), body mass index (BMI), presence of hypertension (blood pressure >140/90 mmHg or requirement for antihypertensive therapy), 24 h urinary protein excretion, serum albumin, creatinine, and uric acid levels, corticosteroid and/or immunosuppressant treatment. Serum albumin, creatinine, and uric acid levels were measured by a Roche MODULAR automatic biochemistry analyzer. Mean arterial pressure (MAP) was defined as diastolic pressure plus a third of the pulse pressure. The estimated glomerular filtration rate (eGFR) was calculated by the Chronic Kidney Disease Epidemiology Collaboration (CKD-EPI) equation adjusted for Asian populations [10]. ESRD was defined as eGFR <15 ml/min/1.73m^2^, initiation of dialysis, or kidney transplantation. Renal endpoint (combined event) contained 50% reduction in eGFR or ESRD. Follow-up data (updated to June 2018) were collected via outpatient and telephone interview by two independent clinical reviewers.

### 2.3. Renal Biopsy

The renal biopsies were performed by the director of Department of Nephrology in our hospital. The biopsy specimens were processed for immunofluorescence, light microscopy, and electron microscopy studies. The diagnostic criteria of IgA nephropathy were as follows: (i) IgA deposited in mesangial for at least 1+ (on a scale from 0 to 4+) in immunofluorescence studies, and the dominant immunoglobulin deposited in the glomeruli was IgA, and (ii) electron-dense deposited in mesangial in EM studies [11].

### 2.4. BC Identification

The Constitution in Chinese Medicine Questionnaire (CCMQ), issued by the China association of TCM in 2009, was adopted to evaluate the participants' BC statuses [4].

All of the participants were asked to complete the CCMQ, which consists of 60 items rated on a 5-point Likert Scale. The scores of all items in each subscale were summarized; then, the sum scores were converted for identifying the participants' BC types. The CCMQ is grouped into nine BC types: gentleness BC, Yang-deficiency BC, Yin-deficiency BC, Qi-deficiency BC, phlegm-dampness BC, dampness-heat BC, blood-stasis BC, Qi-depression BC, and special diathesis BC types. Gentleness BC type is the only balanced BC type, and the other eight BC types are unbalanced BC types.

### 2.5. Statistical Analysis

Patients were stratified by their BC types. Continuous variables were presented as the mean ± standard deviation (SD); categorical variables were presented as a number and percentage. The differences between two groups were compared by the unpaired t-test and *χ*^2^test, as appropriate. The Kaplan–Meier method was used to estimate the renal survival. The relationships between factors and renal survival were assessed with Cox regression. Factors showing statistical significance in univariate analysis were further considered with multivariate analysis. The significance level was* P*<0.05 (two-sided). All statistical analyses were performed using SPSS 17.0 software (SPSS, Chicago, IL, USA).

## 3. Results

### 3.1. Demographic and Clinical Features

We investigated 203 IgA nephropathy patients in this study, with an average age of (40.1±11.6) years, and 111 cases (54.7%) were males. 36.9% of these patients had hypertension. The proteinuria level was (1.68±1.57) g/d, 53.7% of our patients had proteinuria greater than 1.0 g/d, and 11.3% had proteinuria greater than 3.5g/d. Patients were classified into chronic kidney disease (CKD) stages with 43.4% in stage 1, 27.1% in stage 2, 26.1% in stage 3, 3.5% in stage 4, and none in stage 5. The mean follow-up period was (63.9±16.2) months. During follow-up, 32 patients (15.8%) achieved the renal endpoint event. There were 64 participants in the control group, with an average age of (42.4±18.4) years and 34 participants (53.1%) were males.

In this study, the most common BC type was Qi-deficiency BC type; the proportion of Qi-deficiency BC type was 56.2% in the IgA nephropathy group and 37.5% in the control group. The gentleness BC type percentage was lower in the IgA nephropathy group than the control group (*P*=0.027). The proportions of Qi-deficiency BC, phlegm-dampness BC, dampness-heat BC, blood-stasis, and special diathesis BC types were higher in the IgA nephropathy group than the control group (*P* < 0.05) (Table 1). In IgA nephropathy group, 98.0% of cases could be classified for at least one BC type, and 67.5% had more than one imbalanced BC type. In the control group, the percentage was 96.9% for at least one BC type and 55.3% for more than one imbalanced BC type (Table 2).

### 3.2. Distribution of Yang-Deficiency BC

The patients were divided into Yang-deficiency BC group (97, 47.8%) and non-Yang-deficiency BC group (106, 52.2%). At baseline, there were no differences between the two groups in clinical characteristics such as age, BMI, lifestyle behaviors (smoking, alcohol drinking, and less exercise), MAP, initial proteinuria, eGFR, serum uric acid, CKD stage, and corticosteroid and/or immunosuppressant treatment. The male percentage was lower in the Yang-deficiency BC group than the non-Yang-deficiency BC group (*P*=0.011). The serum albumin level was lower in the Yang-deficiency BC group than the non-Yang-deficiency BC group (*P*=0.009). During follow-up, more patients achieved the renal endpoint in Yang-deficiency BC group (23 (23.7%)) than non-Yang-deficiency BC group (9(8.5%),* P*=0.003, Table 3). Yang-deficiency BC was associated with worse renal survival from renal endpoint event (log–rank, 4.84;* P*=0.028).

### 3.3. Distribution of Blood-Stasis BC

The patients were divided into blood-stasis BC group (64, 31.5%) and non-blood-stasis BC group (139, 68.5%). At baseline, there were no differences between the two groups in clinical characteristics such as age, BMI, lifestyle (smoking, alcohol drinking, and less exercise), MAP, initial proteinuria, eGFR, CKD stage, and corticosteroid and/or immunosuppressant treatment. The male percentage was lower in the blood-stasis BC group than the non-blood-stasis BC group (*P*=0.001). The serum albumin level was lower in the blood-stasis BC group than the non-blood-stasis BC group (*P*=0.008). The serum uric acid level was higher in the blood-stasis BC group than the non-blood-stasis BC group (*P*=0.007). During follow-up, more patients achieved the renal endpoint in blood-stasis BC group (18 (28.1%)) than non-blood-stasis BC group (14(10.1%),* P*=0.001, Table 4). Blood-stasis BC was associated with worse renal survival from renal endpoint event (log–rank, 5.96;* P*=0.015).

### 3.4. Risk Factors

Univariate analysis was used to indicate factors associated with an increased risk for the endpoint event (*P*< 0.05). These included initial proteinuria of more than 1g/d, hypertension, renal impairment (eGFR <60mL/min), hypoproteinemia (serum albumin <35 g/L), hyperuricemia (serum uric acid >420*μ*mol/L), Yang-deficiency BC, and blood-stasis BC at the time of renal biopsy. Sex, age, BMI, smoking, alcohol drinking, less exercise, and other seven BC types were not risk factors for disease progression (Table 5). These factors significantly associated with disease progression by univariate analysis were further analyzed in multivariate Cox regression. It identified that only hypertension (hazard ratios (HR) 3.5,* P*=0.009), renal impairment (HR 5.8,* P*<0.001), Yang-deficiency BC (HR 2.3,* P*=0.041), and blood-stasis BC (HR 2.5,* P*=0.017) at biopsy were independent predictors of unfavorable renal outcomes (Table 5).

## 4. Discussion

The BC, appearing first in “Huang Di Nei Jing” (a classics textbook of TCM), is one of the most important characteristics of complementary and alternative medicine [5]. BC is composed of physiologic functions and psychological states [12]. The BC theory has been used to guide disease prevention, healthcare, and medical practice by TCM practitioners for more than 2000 years [13]. Patients with the same disease can be treated differently according to their BC types, which is known as “tong bing yi zhi” in TCM. In addition, the susceptibility and progression of diseases will be different in people with different BC types [2, 14].

In this study, 98.0% of IgA nephropathy patients could be classified for at least one BC type, it confirmed that the CCMQ was suitable for BC identification in IgA nephropathy patients. This study found that the distribution of BC was different between IgA nephropathy patients and normal population; it indicated that BC was associated with the susceptibility of IgA nephropathy; this is consistent with TCM theory. Same as previous study in Hong Kong Chinese populations [15], we found that the most common BC type was Qi-deficiency BC type in IgA nephropathy patients. This study showed that 67.5% of IgA nephropathy patients had more than one imbalanced BC type; overlapping BC types were very common in IgA nephropathy, which showed the implication for TCM individualized treatment.

Same as previous report [4, 16], this study confirmed that male patients were less likely to be Yang-deficiency BC or blood-stasis BC in IgA nephropathy; this is consistent with TCM theory. In the “Yin-Yang” theory of TCM, the male is characterized by “Yang”, while the female is characterized by “Yin”. This study found that the serum albumin level was lower in the Yang-deficiency BC group than the non-Yang-deficiency BC group; this difference was also found in patients with and without blood-stasis BC. In recent years, some studies had indicated the molecular mechanisms and polymorphisms of Yang-deficient BC and blood-stasis BC [16, 17]. Maybe, there were some kinds of corresponding relations between these BC types and the decrease in expression level of certain proteins and require more research. This study showed that the serum uric acid level was higher in the blood-stasis BC group than the non-blood-stasis BC group and require more research.

IgA nephropathy, characterized by IgA deposition in the glomerular mesangium, is not a benign disease [11]. In this study, although many patients were biopsied at a relatively early stage with 70.4% of patients in CKD stages 1 and 2 and none in stage 5, during follow-up, 15.8% of patients achieved the renal endpoint. So, it is necessary to pay more attentions to the risk factors for the progression of IgA nephropathy.

To the best of our knowledge, this is the first report showing that Yang-deficient BC and blood-stasis BC may be independent predictors for the progression of IgA nephropathy. And these new predictive factors are noninvasive and without incurring any costs for BC identification. In TCM, the constitution, inherently forming and developing throughout life, is relatively stable. On the other hand, the constitution is changeable; with the course of diseases lasting, the participants' constitution statuses might change. We will evaluate the participants' constitution types again in the future. In TCM, the constitution is adjustable; therefore, the treatment of tonifying Yang and promoting blood circulation and removing blood-stasis might improve the prognosis for IgA nephropathy patients. Consistent with the previous studies [11, 18], in our study, hypertension was a strong risk factor for the progression of IgA nephropathy. Nevertheless, blood pressure control in China is unsatisfactory; rigorous blood pressure control might improve the prognosis greatly for IgA nephropathy patients [11].

In multivariate analysis, initial proteinuria was not associated with renal outcomes of IgA nephropathy. This finding is contrary to previous reports [18], but similar to Lv's study [11]. In this study, most of our patients had severe proteinuria; 53.7% showed proteinuria greater than 1.0 g/d; 11.3% with proteinuria greater than 3.5g/d. 50.7% of the patients accepted corticosteroid and/or immunosuppressant treatment. Previous report found that patients with mild renal lesions were easy to rapidly enter proteinuria remission [11]. Proteinuria level and remission or not may be partly responsible for these differences. In addition, measuring the time-average proteinuria during follow-up may be more accurate than point proteinuria in baseline for judging the prognosis of IgA nephropathy [18]. A few studies showed that sex, age, BMI at biopsy were also associated with the progression of IgA nephropathy [9, 19, 20]; inconsistent with these previous studies, no such associations were found in our study.

In this study, the histological lesions were not analyzed, for our previous study aimed at renal lesions and found that intrarenal arterial lesions were association with the prognosis of IgA nephropathy [21]. In addition, the limitations of this study included recruitment of participants from a single medical center and lack of BC status evaluating in follow-up.

## 5. Conclusions

Most patients of IgA nephropathy were biopsied at an early stage. Yang-deficiency BC and blood-stasis BC at biopsy were most closely associated with the worse prognosis of IgA nephropathy along with hypertension and renal impairment. Further studies on the role of BC in progression of IgA nephropathy are warranted.

## Figures and Tables

**Table 1:** Distribution of BC in IgA nephropathy group and the control group.

Characteristics	IgA nephropathy group (n=203)	the control group (n=64)	*P* values
Age (years)	40.1±11.6	42.4±18.4	0.352
Male (n (%))	111 (54.7)	34 (53.1)	0.828
Gentleness BC (n (%))	32(15.8)	18(28.1)	0.027
Yang-deficiency BC (n (%))	97(47.8)	23(35.9)	0.097
Yin-deficiency BC (n (%))	80(39.4)	23(35.9)	0.619
Qi-deficiency BC (n (%))	114(56.2)	24(37.5)	0.009
Phlegm-dampness BC (n (%))	90(44.3)	19(29.7)	0.038
Dampness-heat BC (n (%))	93(45.8)	16(25.0)	0.003
Blood-stasis BC (n (%))	64(31.5)	12(18.8)	0.048
Qi-depression BC (n (%))	41(20.2)	10(15.6)	0.417
Special diathesis BC (n (%))	80(39.4)	15(23.4)	0.020
No BC (n (%))	4(2.0)	2(3.1)	0.587

**Table 2:** Number of BC types in IgA nephropathy group and the control group (n (%)).

Number of BC types	IgA nephropathy group (n=203)	the control group (n=64)
0	4(2.0)	2(3.1)
1	62(30.5)	26(40.6)
2	22(10.8)	7(10.9)
3	27(13.3)	14(21.9)
4	19(9.4)	7(10.9)
5	25(12.3)	2(3.1)
6	15(7.4)	2(3.1)
7	19(9.4)	4(6.3)
8	10(5.0)	0(0)

**Table 3:** Clinical characteristics at biopsy and follow-up for patients with Yang-deficiency BC and non-Yang-deficiency BC.

Characteristics	Yang-deficiency BC (n=97)	Non-Yang-deficiency BC (n=106)	*P* values
Age (years)	41.0±10.9	39.2±12.1	0.271
Male (n (%))	44 (45.4)	67 (63.2)	0.011
BMI (kg/m^2^)	24.2±4.0	24.7±3.4	0.386
Smoking (n (%))	20(20.6)	27(25.5)	0.413
Alcohol drinking (n (%))	18(18.6)	24(22.6)	0.473
Less exercise (n (%))	26(26.8)	26(24.5)	0.711
MAP(mmHg)	99.1±13.8	97.3±12.6	0.352
Urinary protein (g/d)	1.78±1.80	1.58±1.32	0.398
Serum albumin (g/L)	37.4±6.0	39.6±5.5	0.009
eGFR (ml/min)	80.2±29.0	80.3±28.7	0.985
Serum uric acid (*μ*mol/L)	374.2±97.6	389.7±103.7	0.275
CKD stage (n (%))			0.989
stage 1	43(44.3)	45(42.5)	
stage 2	26(26.8)	29(27.4)	
stage 3	25(25.8)	28(26.4)	
stage 4	3(3.1)	4(3.8)	
Treatment^*∗*^(n (%))	45(46.4)	58(54.7)	0.236
Follow-up (months)	64.6±17.8	63.4±14.5	0.600
Endpoint event (n (%))	23(23.7)	9(8.5)	0.003

*∗*Corticosteroid and/or immunosuppressant treatments; BMI: body mass index; MAP: mean arterial pressure; eGFR: estimated glomerular filtration rate; CKD: chronic kidney disease; endpoint event: 50% decline in eGFR or end-stage renal disease.

**Table 4:** Clinical characteristics at biopsy and follow-up for patients with blood-stasis BC and non-blood-stasis BC.

Characteristics	Blood-stasis BC (n=64)	Non- Blood-stasis BC (n=139)	*P* values
Age (years)	39.6±10.4	40.3±12.1	0.689
Male (n (%))	19 (29.7)	92 (66.2)	0.001
BMI (kg/m^2^)	24.5±4.3	24.4±3.4	0.955
Smoking (n (%))	12(18.8)	35(25.2)	0.313
Alcohol drinking (n (%))	14(21.9)	28(20.1)	0.777
Less exercise (n (%))	14(21.9)	38(27.3)	0.407
MAP(mmHg)	98.5±13.1	98.0±13.3	0.819
Urinary protein (g/d)	1.92±1.70	1.56±1.49	0.144
Serum albumin (g/L)	37.0±5.8	39.4±5.7	0.008
eGFR (ml/min)	77.1±30.0	81.7±28.2	0.299
Serum uric acid (*μ*mol/L)	410.5±118.7	369.3±89.0	0.007
CKD stage (n (%))			0.739
stage 1	25(39.1)	63(45.3)	
stage 2	17(26.6)	38(27.3)	
stage 3	19(29.7)	34(24.5)	
stage 4	3(4.7)	4(2.9)	
Treatment^*∗*^(n (%))	31(48.4)	72(51.8)	0.656
Follow-up (months)	64.8±17.3	63.6±15.6	0.624
Endpoint event (n (%))	18(28.1)	14(10.1)	0.001

*∗*Corticosteroid and/or immunosuppressant treatments; BMI: body mass index; MAP: mean arterial pressure; eGFR: estimated glomerular filtration rate; CKD: chronic kidney disease; endpoint event: 50% decline in eGFR or end-stage renal disease.

**Table 5:** Factors influencing renal survival from endpoint event by univariate and multivariate Cox regression.

Characteristics	Univariate		Multivariate	
	HR(95%CI)	*P* value	HR(95%CI)	*P* value
Hypertension	6.5(2.9-14.5)	<0.001	3.5(1.4-8.7)	0.009
Renal impairment	10.6(4.4-25.8)	<0.001	5.8(2.2-15.4)	<0.001
Yang-deficiency BC	2.3 (1.1-5.1)	0.033	2.3(1.0-5.2)	0.041
Blood-stasis BC	2.3 (1.2-4.8)	0.019	2.5(1.2-5.2)	0.017
Proteinuria of more than 1g/d	2.4 (1.0-5.4)	0.037		NS
Hypoproteinemia	2.8(1.3-5.8)	0.007		NS
Hyperuricemia	7.3(3.3-15.8)	<0.001		NS

Hypertension: blood pressure >140/90 mmHg or requirement for antihypertensive therapy; renal impairment: eGFR<60 mL/min. Hypoproteinemia: serum albumin <35 g/L; hyperuricemia: serum uric acid >420*μ*mol/L; NS: not significant.

## Data Availability

The data used to support the findings of this study are available from the corresponding author upon request.
